# High vaccine effectiveness against coronavirus disease 2019 (COVID-19) and severe disease among residents and staff of long-term care facilities in Norway, November 2020–June 2021

**DOI:** 10.1017/ash.2021.246

**Published:** 2022-01-17

**Authors:** Jostein Starrfelt, Anders Skyrud Danielsen, Oliver Kacelnik, Anita Wang Børseth, Elina Seppälä, Hinta Meijerink

**Affiliations:** 1 Department of Infection Control and Preparedness, Norwegian institute of Public Health, Oslo, Norway; 2 Department of Microbiology, Oslo University Hospital, Oslo, Norway; 3 Department of Infection Control and Vaccines, Norwegian Institute of Public Health, Oslo, Norway; 4 European Programme for Intervention Epidemiology Training (EPIET), European Centre for Disease Prevention and Control (ECDC), Stockholm, Sweden

## Abstract

Coronavirus disease 2019 (COVID-19) causes high morbidity and mortality in long-term care facilities (LTCFs). COVID-19 vaccine effectiveness against infection was 81.5% and 81.4% among fully vaccinated residents and staff in LTCFs. The vaccine effectiveness against COVID-19-associated death was 93.1% among residents, and no hospitalizations occurred among fully vaccinated staff.

Age is the main factor associated with severe outcomes for coronavirus disease 2019 (COVID-19), and outbreaks in long-term care facilities (LTCFs) have caused a large burden of disease. To open society without excess mortality while preserving healthcare capacity, many countries, including Norway, have prioritized vaccinating residents and staff of LTCFs.^1–3^ This policy depends on a sufficient vaccine effectiveness in this population. Some studies have assessed the effect of COVID-19 vaccination among residents of LTCFs; showing that COVID-19 vaccines reduce both infections and severity of disease.^1,3–6^ However, few have looked at the combined picture presented by staff and residents.

We estimated the effectiveness of COVID-19 vaccines (1) in preventing polymerase chain reaction (PCR)–confirmed severe acute respiratory coronavirus virus 2 (SARS-CoV-2) infections in both residents and staff of LTCFs, (2) in preventing COVID-19-associated hospitalization in staff and (3) in preventing deaths in residents as a proxy for severe disease.

## Determining vaccine effectiveness in long-term care facilities

We obtained data from Beredt C19, a preparedness registry containing individual-level data from various Norwegian registries (Appendix 1 online).^7^ We included data from 6 weeks prior to vaccination start in Norway (December 27, 2020), up to a PCR-positive SARS-CoV-2 test, hospitalization with COVID-19 as primary diagnosis or COVID-19-associated death, death from any cause, or end of follow-up (June 15, 2021). We included all HCWs employed at LTCFs in the third week of January 2021 and residents registered with a long-term stay at an LTCF in 2020. We excluded those with prior SARS-CoV-2 infection and individuals for whom the interval between doses were less than national absolute minimums (ie, 22 days for the Spikevax/Moderna vaccine, 21 days for the Vaxzevria/AstraZeneca vaccine, and 19 days for the Comirnaty/Pfizer/BioNtech vaccine).^7^ Underlying conditions were categorized as high risk or medium risk as described by the national vaccination program.^7^ Vaccination status was divided in to 3 categories: unvaccinated (unvaccinated or <14 days after the first vaccine dose), partially vaccinated (≥14 days after the first vaccine dose or <7 days after the second vaccine dose), and fully vaccinated (≥7 days after the second vaccine dose).

We used Cox proportional hazard models to estimate vaccine effectiveness by modelling COVID-19 vaccination status as a time-varying covariate, adjusting for age, sex, and underlying conditions. Vaccine effectiveness was calculated as 1 minus the hazard ratio (1–HR) with corresponding 95% confidence intervals (CIs). We included 31,489 residents (median, 87 years; IQR, 81–92) at a total of 819 institutions, of whom 26,905 (85.4%) received at least 1 dose during the follow-up period compared to 63,000 (71.1%) of the 88,549 HCWs (median, 39 years; IQR, 27–53). Of 27,067 residents, 27,038 (99.9%) received the Comirnaty vaccine (Pfizer/BioNTech; BNT162b2). Among 68,714 HCWs, and 39,622 (57.7%) received the Comirnaty vaccine and 26,210 (38.1%) received the Vaxzevria vaccine (AstraZeneca; ChAdOx nCoV-19; AZD1222), either 2 doses or in combination with an mRNA vaccine.

**Fig. 1.**
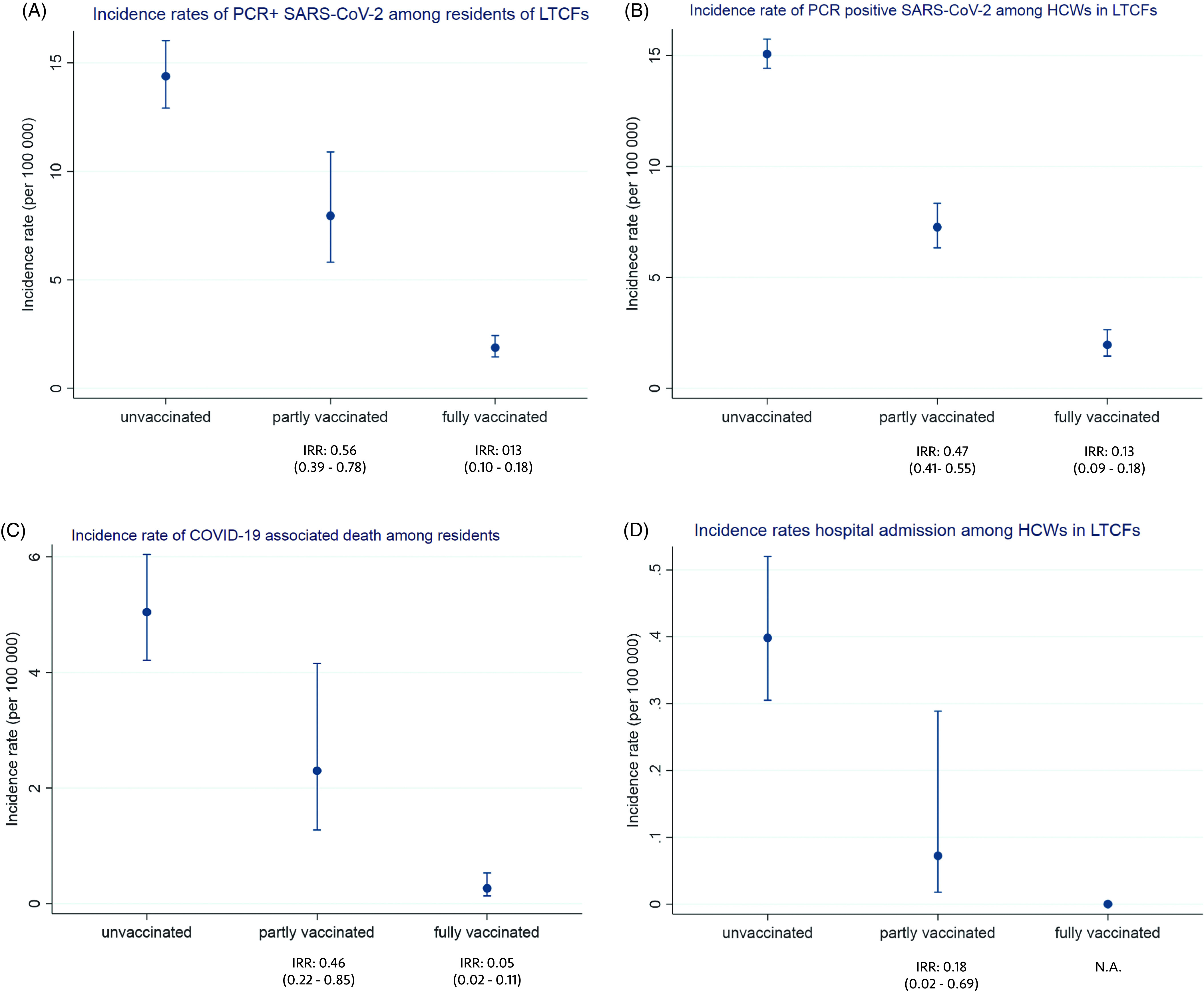
Incidence rates per 100,000 person days with 95% confidence intervals by vaccination status among residents and healthcare workers in long-term care facilities (LTCFs): PCR-positive SARS-CoV-2 infection among residents (A) and staff (B), COVID-19-associated death among residents (C), and hospital admissions with COVID-19 as main cause among healthcare workers (D). Note. Unvaccinated was defined as unvaccinated or <14 days after the first dose; partially vaccinated was defined as ≥14 days after the first dose to <7 days after the second dose; and fully vaccinated was defined as ≥7 days after the second dose. IRR: incidence rate ratio compared to unvaccinated.

The incidence rates of COVID-19 were associated with vaccination status in both residents and HCWs (Fig. 1). The overall adjusted vaccine effectiveness against SARS-CoV-2 infection including residents and HCWs was 81.0% (95% CI, 76.5%–84.6%) for those who were fully vaccinated and 40.8% (95% CI, 31.8%–48.5%) for those who were partially vaccinated.

## Residents of long-term care facilities

Vaccine effectiveness against PCR-positive SARS-CoV-2 was 81.5% among fully vaccinated residents. Sex, age, and underlying conditions had limited effect on the estimates, probably due to the relatively uniform population characteristics (Table 1).

**Table 1.** Estimated COVID-19 Vaccine Effectiveness (VE) Against Laboratory-Confirmed SARS-CoV-2 Infection, Hospitalization With COVID-19 as Main Cause (Staff), and COVID-19-Associated Death (Residents) Among Residents and Staff of Long-Term Care Facilities

Vaccine Status	Events	Person Time, Days	Rate^a^	Crude		Adjusted^b^	
				VE	95% CI	VE	95% CI
**Residents**							
**PCR-positive SARS-CoV-2**							
Unvaccinated	332	2,338,969	14.2	Reference		Reference	
Partially vaccinated	38	478,310	7.9	−20.4	−69.8 to 14.6	−20.4	−70.0 to 14.7
Fully vaccinated	57	3,015,338	1.9	81.7	75.6–86.3	81.5	75.3–86.1
**COVID-19-associated death**							
Unvaccinated	118	2,338,969	5.0	Reference		Reference	
Partially vaccinated	11	478,310	2.3	14.8	−69.6 to 57.2	22.7	−52.6 to 60.8
Fully vaccinated	8	3,015,338	0.3	90.4	76.6–95.5	93.1	85.1–96.8
**Healthcare workers**							
**PCR-positive SARS-CoV-2**							
Unvaccinated	2040	13,562,011	15.0	Reference		Reference	
Partially vaccinated	197	2,771,252	7.1	47.3	38.7–55.7	45.0	35.3–53.2
Fully vaccinated	42	2,148,238	2.0	83.0	76.7–87.6	81.4	74.5–86.4
**Hospital admission with COVID-19 as main cause**							
Unvaccinated	54	13,562,011	0.4	Reference		Reference	
Partially vaccinated	2	2,771,252	0.1	80.1	14.2–95.4	81.7	21.0–95.8
Fully vaccinated	0	2,148,238	NA	NA	NA	NA	NA

Note. CI, confidence interval. Note. Unvaccinated was defined as unvaccinated or <14 days after the first dose; partially vaccinated was defined as ≥14 days after the first dose to <7 days after the second dose; and fully vaccinated was defined as ≥7 days after the second dose. PCR, polymerase chain reaction assay; NA, not available.

a per 100,000 person days.

b Adjusted for sex, age, and underlying conditions.

In Norway, residents of LTCFs often receive health care at the facility and are not admitted to hospitals unless it is in the best interest of the resident. This population has the highest COVID-19 mortality, so we estimated the vaccine effectiveness against COVID-19-associated death in this population. During the study period 6,710 residents died, of whom 137 had COVID-19. The vaccine effectiveness against COVID-19-associated death was 93.1% among fully vaccinated individuals (Table 1).

## Healthcare workers in long-term care facilities

Among HCWs, the vaccine effectiveness against PCR-positive SARS-CoV-2 was 45.0% among those who were partially vaccinated and 81.4% among those who were fully vaccinated, adjusting for age, sex, underlying conditions, and calendar time (Table 1). In Norway, COVID-19-associated mortality in the general population has remained low; therefore, we used hospital admissions with COVID-19 as primary diagnosis as a measure of disease severity in HCWs. Overall, 56 individuals were hospitalized with COVID-19 as their primary diagnosis, of whom 2 were among partially vaccinated and none were fully vaccinated. The adjusted vaccine effectiveness against COVID-19 hospitalization was 81.7% for partially vaccinated HCWs in LTCFs (Table 1).

## Discussion

Vaccine effectiveness was high against both SARS-CoV-2 infection and severe disease in fully vaccinated residents and HCWs in LTCFs in Norway. Importantly, 1 vaccine dose protected well against hospital admission among HCWs. Our estimates coincide with those from other studies in similar settings with high disease burden such as LTCFs.^1–6,8,9^ In a pragmatic and rapid review, Salcher-Konrad et al^10^ listed other studies showing similar levels of protection among residents, although these reported somewhat lower protection against infection than our estimates. Only Moustsen-Helms et al^1^ also estimated vaccine effectiveness against infection for HCWs in the same study, and they reported similar protection after the second dose. Thus, our findings gave a more complete picture of vaccine protection in these institutions by combining vaccine effectiveness against both infection and disease and by including both residents and workers.

Residents of LTCFs were identified as a very vulnerable population and were prioritized for vaccination in Norway. Vaccination roll-out in this group was quick (90% had received their first dose by 20 January),^7^ thus residents spent limited time as partially vaccinated as most residents received their second dose 3 weeks after the first; thus, follow-up time was limited in this group.^1,4^ This factor could explain why, despite significantly lower incidence rates, we could not show protection among partially vaccinated residents. Vaccination might have been withheld for medical reasons (eg, frailty or a history of severe allergic reactions to vaccines), and those individuals might also have had the highest risk of severe outcomes after COVID-19, which could have biased the vaccine effectiveness estimate upward. However, this bias seems to be limited, as can be seen from the lack of effect of age, sex, and underlying conditions on vaccine effectiveness.

Protection against COVID-19 hospitalization in HCWs was high after just 1 dose of vaccine. The number of hospital admissions among HCWs was low but was even lower after vaccination. Furthermore, with fewer (undetected) infections among staff, the total protection for (unvaccinated) residents is potentially increased through staff vaccination. The effect of 1 dose also underscores the importance of ensuring that temporary staff have received 1 dose of vaccine prior to working in an LTCF.

This study had several limitations. These results may not be generalizable to settings with a longer or more heterogenous interval between doses. At the beginning of the study period, only a small proportion of samples had been confirmed as the α variant (B1.1.7). This became the dominant circulating strain in Norway from early February and was replaced by the δ variant (B.1.617.2) by July 2021.

The outcomes of this study are essential for the guidance of COVID-19 measures in LTCFs. Our results showcase the importance of quickly achieving full protection among the frail and elderly as well as the effectiveness of 1 dose in healthcare workers. The pandemic has restricted the life of residents in LTCFs. Through better knowledge, we can adjust COVID-19 guidelines for residents in LTCFs to fewer, targeted measures that can be taken, which might contribute to better quality of life. This knowledge is transferable to similar situations, like influenza, where vaccination of both residents and HCWs is of particular importance.
